# Surrogacy and Adoption: An Empirical Investigation of Public Moral Attitudes

**DOI:** 10.1007/s11673-024-10343-1

**Published:** 2024-03-29

**Authors:** T. Baron, E. Svingen, R. Leyva

**Affiliations:** 1 Philosophy Department, University of Nottingham, School of Humanities, University Park, Nottingham, NG7 2RD United Kingdom; 2 Department of Social Policy, Sociology and Criminology, University of Birmingham, Muirhead Tower, Ring Road North, Birmingham, B15 2TN United Kingdom

**Keywords:** Surrogacy, Adoption, Fertility, Public values, Families

## Abstract

Surrogacy and adoption are both family-making measures subject to extensive domestic and international regulation. In this nationally representative survey study (N = 1552), we explore public attitudes to various forms of surrogacy and adoption in the United Kingdom, in response to an early proposal to allow “double donor” surrogacy as part of the ongoing legal reform project. We sought to both gauge public moral support for adoption and surrogacy generally, the effect that prospective parents’ fertility had on this support, and the extent to which the public would find equivalencies between “double donor” surrogacy (DDS) and planned private adoption (PPA) to be morally significant. Our findings indicate that whilst there is broad baseline support for all forms of adoption and surrogacy, this support increases significantly when one or both prospective parents are infertile. These findings also suggest that the language in which a family-making arrangement is characterized has a greater influence on moral support for the arrangement than practical features such as the biological relationship (or absence thereof) between one/both parents and the child.

## Introduction

“Surrogacy” is the name usually given to arrangements in which a woman agrees to conceive and give birth to a child who a different individual or couple will then parent. One or both intending parents contribute their sperm or eggs to the process and are, therefore, the genetic parents of the resulting child. A surrogate mother may provide “gestational” surrogacy (in which she gestates a child with no genetic relationship to her) or “traditional” surrogacy (in which the surrogate mother uses her own egg). Which version is undertaken will depend on the laws of the country in question, the forms of clinical assistance available, and the fertility of the intending parents. In some countries, surrogacy is entirely illegal; in others, these arrangements exist in a grey area, neither illegal nor regulated; and in others, the practice is legal, recognized, and regulated by the State (Shephard 2021). The nature and ethical permissibility of surrogacy, and the distinctions between surrogacy and adoption, have been long-standing subjects of philosophical, ethical, and legal debate for decades. Philosophers continue to disagree regarding the manner in which moral rights and obligations to children are acquired and dispensed with, the moral significance of biological parenthood, whether surrogacy (paid or unpaid) commodifies children and/or the body of the surrogate mother, and how various interests should be weighed in disputes over custody and child-rearing (Baron 2023b, 108–127).

The growth in popularity of surrogacy, together with several problem cases arising in international and domestic surrogacy in recent years, have given rise to increasingly pressing debates about legal regulation of the practice. A number of countries have modified their surrogacy laws in recent years to ban reproductive tourism and/or commercial surrogacy in response to either concerning trends or specific problem cases (Pande 2021; Piersanti et al. 2021). These include cases in which, for example, commissioning parents have divorced during a surrogate mother’s pregnancy and refused to collect the child at birth or have reneged on a surrogacy agreement following the discovery of foetal abnormalities (Schover 2014; Choudhury 2016). Calls for legal reform in other countries also came in the wake of large numbers of newborns left without legal parents in Ukraine when the COVID-19 pandemic and then Russia’s invasion of the country prevented commissioning parents from coming to collect them from other parts of the world (König 2023).

The United Kingdom is among those countries considering a legal overhaul; the Law Commission set out its recommendations for reform in a draft Bill published in March 2023, following a lengthy period of consultation and policy development. The focus of this paper is one proposed reform that was *not* ultimately included in the draft Bill: to allow so-called “double donor” surrogacy (DDS). We build upon earlier work exploring the similarities of this practice to private planned adoption. Of the various ethical debates surrounding surrogacy, that of central concern to our project is concerned with the conditions under which custody—and children themselves—can be transferred between parents. The transfer of children and (moral) rights and responsibilities over those children is widely taken as justified only by the best interests of those children and not by the desires of either original or would-be parents. As we observe in the next section, this has resulted in different ethical responses to surrogacy and adoption within philosophy of parenthood.

In light of the Law Commission’s reform project, there is a clear and pressing need to canvass public moral support—for both new and existing policies. In this paper, we present the results of an empirical study gauging the attitudes of a representative sample of the United Kingdom's population, with regard to adoption and surrogacy arrangements carried out by hypothetical couples. Although surrogacy arrangements are utilized for family-making by couples of many sexualities, and indeed by single people, the hypothetical scenarios used in our study presented only heterosexual couples. As we explain further in our methodology section, this was considered necessary in order to constrain the number of variables influencing the moral attitudes and intuitions of participants in response to various possible forms of surrogacy and adoption. Our aims were twofold: First, we wanted to investigate participants’ moral attitudes towards surrogacy and adoption and the correlation of these with demographic factors such as age, education, and parenthood. Second, we wanted to investigate how the public picked out morally relevant facts: would they express consistent moral attitudes towards the same kind of arrangement described using different terminology?

In the paper’s third section, we explicate our findings and discuss their implications. One key finding gleaned from our data is that differences in framing often obfuscate important equivalencies between hypothetical scenarios. The use of specific terms associated with clinical assisted reproduction produced a pronounced difference in attitudes towards practically equivalent scenarios. For example: a larger proportion of lay people expressed support for arrangements in which a couple asked others to produce a biologically unrelated child for them to rear when these arrangements were described using the words “donor” and “surrogate” to refer to the child’s biological parents, than when identical arrangements were described using simpler, non-clinical descriptions (see sections "Results" and "Discussion and Limitations"). Given the importance of analogy as a philosophical tool—whether in prompting moral intuitions or seeking reflective equilibrium—this should inform how we use this tool across the board. However, it also has implications for the way in which polling (and other means of gleaning public morality) is carried out in future projects.

We should note here that by “public morality” we mean a largely shared moral attitude across a coherent population group, as distinct from morality per se, the latter of which may be considered independently of historical and social context. Assuming a non-relativist view of morality, it follows that both the law and public morality may be inconsistent with morality per se. But of course, any law or regulation fundamentally at odds with widespread public morality will be decidedly unpopular and, frankly, difficult to enforce.

Some laws have little (if anything) to do with morality. The legal requirement to drive on the left-hand side of the road in the United Kingdom is not grounded on any belief that the left is morally superior to the right, nor (we imagine) could anyone feel that this legal requirement comes into conflict with their own personal moral principles. The relationship between law and morality is therefore a little more complicated than the Warnock Committee’s claim (that the law “is the embodiment of a common moral position”) might suggest. There is, nevertheless, a widely shared belief that laws with moral content should be subject to moral scrutiny; this belief is sufficient to motivate an investigation into public moral attitudes regarding actual and potential policy in the area of family-making.

## Adoption and Surrogacy in Ethics and Philosophy of Parenthood

Long-standing debates regarding the ethical permissibility of surrogacy have various concerns at their centre. In the context of philosophy of parenthood and reproductive ethics, a key part in these debates is played by *moral rights and responsibilities* over children, the means by which people become parents, and whether people dispose with their moral rights and responsibilities legitimately. The comparison between surrogacy and adoption in this field is relevant precisely because the differential ethical treatment some philosophers grant surrogacy and adoption (with regard to, for example, the permissibility of private and/or commercial arrangements) hinges upon whether prospective parents are taken to have an independent moral right to parent the child in question. This rests on accounts of moral parenthood that may be applied in *general*; there may of course be further ethical issues regarding the particulars of specific cases (for example, whether a surrogate mother or first family is coerced into giving up a child or lacks appropriate support to make their decision autonomously).

A common framing of surrogacy in philosophy of parenthood presents this as a form of assisted reproduction by which a couple can have “their own” child—that is, without the exchange or transfer of moral rights and responsibilities regarding the child. However, some philosophers have argued that surrogacy is akin to (or even a sub-category of) adoption, a view with important implications for the permissibility of private and/or commercial arrangements (see for example Gheaus and Straehle 2024; Steinbock 1988; Robertson 1983). Consider Velleman’s argument:

[W]e don’t think that adults are permitted to conceive a child with the prior intention to put it up for adoption. A woman may not decide to conceive simply in order to have the experience or health benefits of pregnancy, we think, no matter how confident she may be of finding suitable adoptive parents to take over her subsequent responsibilities. Thus, we regard parental obligations as transferable, morally speaking, only under exigencies that make their transfer beneficial for the child rather than convenient for the parents. (Velleman 2008, 252)

If surrogacy were understood as a form of pre-planned adoption, then the above criticism would seem to apply clearly to surrogacy. This potential result has itself been taken by some as a reason not to accept this framing. Krimmel, for example, argues that surrogacy *cannot* be a kind of adoption precisely because that would make this practice both strange and morally dubious:

At first blush [surrogacy] looks to be little different from the typical adoption, for what is an adoption other than a transfer of responsibility from one set of parents to another? The analogy is misleading, however, for two reasons. First, it is difficult to imagine anyone conceiving children for the purpose of putting them up for adoption. And, if such a bizarre event were to occur, I doubt that we would look upon it with moral approval. (Krimmel 1983, 36)

Of course, Krimmel seems to presuppose here that adoption takes only one form: the transfer of children into the broader adoption system without specific parents in mind. The more reasonable analogy would be between surrogacy and *private* adoption agreements, in which rearing parents may be chosen long before the child is born.^1^ This brings us to certain key legal differences between adoption and surrogacy as currently allowed in the United Kingdom. One crucial difference is that whilst it is a criminal offence to ask any private individual or organization to provide a child for adoption (or offer to do so), there is no equivalent prohibition against asking a private individual or organization to provide one with a child through surrogacy, or against offering to be a surrogate mother (Adoption and Children Act 2002 s. 92[1-2]). In other words, whilst private adoption arrangements are illegal, private surrogacy arrangements are not. We explore this distinction, and its role in motivating our study, in the next section.

## Surrogacy and Private Adoption in U.K. Law

The United Kingdom is one of a few European countries that allows surrogacy and provides a framework through which these arrangements can be legally accommodated and processed. Whilst adoption and surrogacy are processed differently and regulated by distinct sets of laws, there is a significant overlap between the two. One overlap, for example, is in the default attribution of legal parental responsibility (PR) to a child’s gestational mother (and to her spouse/civil partner, if applicable). This is the case regardless of any existing surrogacy arrangement or any pre-birth agreement to give up the child for adoption or surrogacy purposes. With her consent, the birth mother’s PR may currently only be terminated by court order. This consent can only be given after six weeks have passed since the child’s birth. Neither adoption nor surrogacy arrangements are currently legally enforceable. The last decade, in particular, has seen calls for reform from various parts of the political spectrum: concerns regarding the ex-poste parental order application system, for example, have given rise to criticism of the United Kingdom as allowing commercial surrogacy “through the back door.” From other quarters, however, the default attribution of PR to surrogate mothers has been criticized as disrespectful to the autonomous wishes of both surrogates and commissioning parents, and as miring the process in uncertainty for the latter.

Following a lengthy consultation and policy development process, the Law Commission of England & Wales and the Scottish Law Commission have produced a draft Bill outlining specific recommendations for reform.^2^ One such reform reverses the current default attribution of legal parental status in the case of surrogacy. For intending parents and surrogate mothers following the “New Pathway,” the need to apply for a parental order would be eliminated. Instead, the intending parents would be legal parents of the child from birth and the gestational mother would have no parental responsibilities or rights. However, one proposed reform that we found to be of particular interest, although outlined in the original consultation paper, was left out of the draft Bill. This was the proposal to eliminate the (current) requirement for a genetic link between the child and at least one intending parent in surrogacy arrangements. The authors of the consultation report suggested that so-called “double donation” surrogacy (DDS) could be allowed in cases in which the intending parents (or single intending parent) were clinically infertile and therefore unable to use their own egg/sperm to have a child through normal surrogacy. DDS would involve the conception of a child by a surrogate mother using donor sperm *and* either a donor egg (or the surrogate’s egg). The resulting child would therefore have no biological relationship with the individual or couple who commissioned her.

As has been argued in more detail elsewhere, DDS shares important equivalencies with private planned adoption (Baron 2023a). Private planned adoption (hereafter PPA) is the planned conception, birth, and transfer of a child to prospective parents, arranged by the relevant parties privately (that is, without the mediation of a registered agency or other regulatory body acting for the child’s best interests). As noted above, private adoption is currently illegal in the United Kingdom per the Adoption and Children Act 2002. The primary legal distinction between surrogacy and private adoption is found in the requirement for at least one intended parent to have a genetic link to the child (via their contribution of egg/sperm) in surrogacy, this being one condition of the granting of a parental order. Pre-conception intentions regarding parenthood are irrelevant to the attribution of legal parenthood at a child’s birth. As noted in the Law Commission’s consultation document, the difficulty of distinguishing between DDS and private adoption, should this proposal be carried through, was therefore one of the key concerns raised against the proposal during the consultation process.

Private planned adoption could theoretically take many forms. A more “clear-cut” case would involve an individual or couple simply asking another couple to conceive a child through sexual intercourse and then handing it over to them to raise as their own. However, PPA could also involve (for example) an individual or couple asking a friend or stranger to conceive using at-home artificial insemination with donor sperm, or at a clinic using a donor embryo. The manner of a child’s conception (that is, whether through sexual intercourse or not) does not currently make a legal difference to whether an arrangement counts as adoption or not.

However, the terminology used to describe the arrangement, and perhaps the intervention of clinical practitioners, might allow the relevant relationships to be reframed. The scenario in which a couple or individual asks another individual to act as a surrogate mother, and to conceive using donor sperm or a donor embryo, is described by the authors of the consultation report as “double donation surrogacy” (DDS) rather than as PPA. The vital point to note, however, is that the arrangement would be practically equivalent to PPA in important ways: in particular, the absence of a biological relationship between the intending parents and the child, and the shared intentions of the parties that the child be conceived “for” and given over to those intending parents. Any such arrangement would, under current U.K. law, be categorized as a private adoption and would (as such) be a criminal offence. We hypothesize that the description of an arrangement using the terminology of DDS, rather than that of PPA, makes a difference to the public’s moral attitude towards the arrangement. We also expect that associated framings of adoption/surrogacy scenarios—particularly the family and fertility circumstances of an individual or couple seeking to have a child—will influence public attitudes regarding the moral permissibility of the relevant course of action.

This is not to say that the family and fertility circumstances of those pursuing adoption and surrogacy are the only ethically relevant points of note. The circumstances of the surrogate mother/first family of a child are also highly important and are rightly the central focus of a large body of ethical work on family-making. However, given the research aims of this study, the testing material was constrained to descriptions of surrogacy/adoption arrangements in quite abstract terms. Just as (as noted above) we chose to present only heterosexual couples in order to neutralize the potential influence of homophobic inclinations on participants’ moral judgements, we also omitted to include information about the gestational mother/first family of the child in each hypothetical scenario.

## Methodology

The aim of our study was to capture public moral attitudes and intuitions using quantitative methods involving an online questionnaire with randomized hypothetical scenarios. The participants were informed that they could withdraw at any point before clicking “submit” and only the participants with a completion rate of 100 per cent were included in the analysis. We asked standard demographic questions about age, gender, education, political orientation, and relationship but collected no identifying information, resulting in an anonymised data set.

### Participants

We collected data from a representative stratified sample from 1552 United Kingdom residents, ages eighteen and above (M = 44.8, SD = 16.5 years). The surveying agency Qualtrics was commissioned to recruit participants during February 2023. From 1552 participants, 819 (52.8 per cent) identified as female, 727 (46.9 per cent) identified as male, and 4 (0.3 per cent) identified as non-binary/third gender. 474 (30.5 per cent) reported that they were single, 105 (6.8 per cent) that they were divorced, and 973 (62.7 per cent) as married or cohabiting.

427 (27.5 per cent) participants described themselves as left-wing/liberal, 800 (51.6 per cent) as moderate/centrist, and 325 (20.9 per cent) as right-wing/conservative. 164 (10.6 per cent) were educated to GCSE or equivalent, 164 (10.6 per cent) to A-level qualification or equivalent, 243 (15.7 per cent) to Higher National Certificate or Diploma, 407 (26.2 per cent) to Undergraduate University Degree, and 574 (36.9 per cent) to Postgraduate Degree. This sample group therefore had a non-representative weighting towards higher education: more than twice as many participants were educated to Higher National Certificate/Diploma level or above in this sample than would be in a sample representative of the broader U.K. population.

### Hypothetical Scenarios

To test public attitudes towards different forms of adoption and surrogacy (in the abstract) in combination with different fertility-related circumstances, we presented participants with variations of a hypothetical scenario. In their form, all scenarios present a situation where a heterosexual couple would like to have a child without going through pregnancy and childbirth.^3^ The hypothetical couple had four possible sets of circumstances: a) fertile with children; b) fertile without children; c) one partner infertile; and d) both partners infertile. The way in which the couple wanted to have a child was also split into four options: a) regular adoption; b) using a surrogate mother and sperm/egg from the intending father *or* mother; c) using a surrogate mother and donated sperm *and* egg, and d) “clear-cut” planned private adoption. The last two options thus represented an equivalent outcome—a pre-conception agreement with a third party to produce a biologically unrelated child for the prospective parents—but one was DDS-coded whilst the other was PPA-coded (see section "Surrogacy and Private Adoption in U.K. Law"). Therefore, there were sixteen scenarios available, out of which every participant would be presented with four, distributed randomly. Each scenario received approximately four hundred responses (Table 1, 2 and 3).

**Table 1:** Summary of the scenario versions

Surrogacy/Adoption type (columns)	*Adoption (standard, state-regulated)*	*Surrogate mother and egg/sperm from one partner*	*Surrogate mother with donated egg and sperm*	*Private planned adoption*
Fertility issue (rows)				
*Both fertile, have children*	1.1	1.2	1.3	1.4
*Both fertile, no children*	2.1	2.2	2.3	2.4
*One partner infertile*	3.1	3.2	3.3	3.4
*Both partners infertile*	4.1	4.2	4.3	4.4

**Table 2:** Percentage of participants that responded “agree” or “strongly agree”

Surrogacy/Adoption type (columns)	*Adoption (standard, state-regulated)*	*Surrogate mother and egg/sperm from one partner*	*Surrogate mother with donated egg and sperm (DDS)*	*Private planned adoption*
Fertility issue (rows)				
*Both fertile, have children*	1.1 87.5%	1.2 65.2%	1.3 55.9%	1.4 49.5%
*Both fertile, no children*	2.1 87.4%	2.2 63.4%	2.3 58%	2.4 47%
*One partner infertile*	3.1 97.6%	3.2 87.2%	3.3 83.5%	3.4 70.6%
*Both partners infertile*	4.1 95.9%	4.2 90.2%	4.3 89.7%	4.4 68.5%

**Table 3:** Percentage of participants that responded “agree” or “strongly agree” on the hypothetical scenarios

	*Both fertile, have children*	*Both fertile, no children*	*One partner infertile*	*Both partners infertile*
*Left-wing*	71.9%	70.9%	88.52%	89.7%
*Moderate*	62.75%	61.9%	84.75%	85.5%
*Right-wing*	60.0%	60.6%	79.7%	83.1%
	chi^2^_(2)_ = 14.1574 *p* = .001 *V* = .0955	chi^2^_(2)_ = 12.1702 *p* = .002 *V* = .0886	chi^2^_(2)_ = 11.1268 *p* = .004 *V* = .0847	chi^2^_(2)_ = 7.3529 *p* = .025 *V* = .0688

For every question, the participant was presented with a basic scenario of “[name] and [name] have [family circumstance]. They decide to [surrogacy/adoption type].” The participants were then asked to what extent they agreed that the couple should be allowed to acquire a child in the proposed way. The answers available comprised a 4-point Likert scale ranging from “completely disagree” to “completely agree.” The middle point of “neutral” was omitted, forcing the participants to express an opinion. This design allowed us to measure variation in public support for different policies and the influence that family circumstances and fertility issues had on moral attitudes towards these practices.

## Results

The participants’ responses to the questionnaire indicated that both the family/fertility circumstances of the hypothetical couple (that is, their fertility and whether they had children already) *and* the proposed form of adoption or surrogacy influenced participant attitudes regarding the permissibility of the proposed course of action. The results of support for all the scenarios are presented in the table below.

We ran Pearson’s chi^2^ analyses on the distribution of responses split into binary outcomes of “agree” vs “disagree” against all versions of the scenarios. We conducted distribution tests comparing the support for different policy options: for both fertile with children condition (Pearson chi^2^_(3)_ = 143.8460; *p* < .001; Cramer’s V = 0.3044), both fertile no children condition (Pearson chi^2^_(3)_ = 152.0341; *p* < .001; Cramer’s V = 0.3130), one partner infertile (Pearson chi^2^_(3)_ = 110.3085; *p* < .001; Cramer’s V = 0.2666), and both partners infertile (Pearson chi^2^_(3)_ = 140.3845; *p* < .001; Cramer’s V = 0.3008). The results suggest that support for the presented policies differed by fertility and parental condition.

In hypothetical scenarios in which one or both partners in the couple were infertile, participants expressed overwhelming support for most types of adoption/surrogacy. This includes a PPA-coded scenario, in which the fictional couple asks another couple to conceive a child and hand it over to them to raise. In the scenarios where the couple had no fertility issues, support for any use of surrogacy decreased (and likewise for any use of adoption, although by a smaller margin). The lowest levels of support are shown for clear private adoption scenarios where a couple experiences no fertility issues. Still, even those scenarios received agreement from around half of the participants, with the level of support increasing to 69 per cent to 71 pe rcent in case of fertility difficulties. Levels of moral agreement also increase for the DDS-coded scenario in the presence of infertility (56 to 58 per cent for fertile couples and 84 to 90 per cent for infertile couples).

We were also interested to know whether this issue is something that is split along party lines and conducted an analysis of comparing the self-identified political affiliation with the levels of support for the proposed policies. Results are summarised in the table below.

Consistently with the results presented earlier, fertility issues of the couple remain the most definitive factor. While in the case of a fertile couple, participants who identified as left-wing were 10 per cent more likely to support any adoption policy. However, once both partners in the hypothetical scenario were identified as infertile, the difference dropped to less than 5 per cent. The strength of the association between political orientation and their support of adoption practices is relatively weak, as shown by all Cramer V values below 0.1. These results suggest that political orientation has little effect on the decision to support specific adoption/surrogacy policies.

## Discussion and Limitations

### General Support

As noted above, the study’s results suggest—in line with our starting assumptions—that both the family/fertility circumstances of the hypothetical couple *and* the proposed form of adoption or surrogacy influenced participant attitudes regarding the permissibility of the proposed course of action. In hypothetical scenarios in which one or both partners in the couple were infertile, participants expressed overwhelming support for most types of adoption; this extended to the “clear-cut” cases of private adoption. Our results indicate widespread support for the legal status quo, under which couples may use surrogacy to have a child with one or both of their own gamete(s). 63 to 65 per cent of participants supported this policy in the case of a fertile couple, and 87 to 90 per cent supported the policy in cases in which one or both of the prospective parents was described as infertile. The lowest levels of support were indicated for clear-cut planned private adoption scenarios where the hypothetical couple experienced no fertility issues; however, even these scenarios received support 47 to 50 per cent of the time, rising to 69 to 71 per cent in case of infertility in one or both partners in the scenario.

There are two implications we can draw from these results. The first is that moral attitudes towards a family-making or reproductive practice are not solely based on the biological relationships (or lack thereof) between the child and intended parents and the intentions with which the child is conceived. Given the practical equivalencies between DDS and PPA, if that were the case, then these scenarios would be expected to give rise to equivalent responses. The language used to describe a practice thus appears to influence public moral attitudes towards that practice (see section "Impact of Language and the Future of Public Polling"). The second implication we can draw from the above is that when biological barriers to natural conception exist, support for any form of adoption or surrogacy arrangement rises—including support for “normal” adoption. Most of the public surveyed showed higher levels of support for any policy allowing a couple experiencing infertility to acquire a child, including in the case of the two policies that are prohibited by current U.K. law.

In seeking explanations for this second trend, we hypothesize two possible directions. The first refers to both widely held normative beliefs about the place of family-building in “standard” life pathways and the potential influence of empathy. It may be that participants were more likely to express support for adoption or surrogacy for infertile couples because they view parenthood as a part of the life cycle that should be attainable by all, and/or further, may feel empathy for those who are barred from achieving this through unassisted procreation. A second way in which we might interpret consistently increasing support for adoption/surrogacy in response to infertility might be by reference to public understandings of adoption/surrogacy systems and the availability of children. If adoptive or surrogate-born children are seen as a scarce resource, it may be that those who are fertile are seen as less “in need” or deserving of access to such services than those who are infertile. However, further and more detailed research would be needed to ascertain more clearly the source of this trend (see section "Expansion for Future Research").

### Political Affiliation

We did not find evidence to suggest that public attitudes regarding different kinds of family-making processes are strongly affected by political affiliation. The biggest split along the party lines was seen in the scenarios with fertile couples; here, differences in the likelihood of support were no greater than 10 per cent. A caveat here must be that, as noted above, our study restricted hypothetical scenarios to include only randomized fictional *heterosexual* couples. We can, therefore, at most, suggest that our results indicate no connection between political affiliation and attitudes towards adoption as compared to surrogacy, surrogacy as compared to DDS, and so on; however, it would be implausible to claim that political affiliation has no influence on attitudes towards adoption, assisted reproduction, and family-making *at all.*

This is nevertheless an important finding for the future of policy discussions around the issue of family-making—not only regarding the current surrogacy reform project but also with regards to further policy-making projects—as it would suggest that “base” policy can be taken as a party-neutral issue.

### Impact of Language and the Future of Public Polling

A key aim of the questionnaire was to determine whether the manner in which an adoption/surrogacy arrangement was described to the public affected their level of support for the particular policy. We were particularly interested in differences in the response to DDS-framed scenarios and PPA-framed scenarios. DDS is equivalent to private planned adoption in important practical respects (see sections 1.2-1.3). Nevertheless, the results indicated that—across scenarios in which a couple asked someone else to provide a biologically unrelated child for them—many of the participants perceived those described using DDS-framed terminology (such as “surrogate” and “donor egg/sperm”) to be different to those described using PPA-framed terminology. Specifically, DDS-framed scenarios received consistently higher moral support from participants than PPA-framed scenarios. This finding aligns with our hypothesis regarding the importance of language in communicating policy and support for such policy: asking someone if a couple should be allowed to commission the procreation of an unrelated child for them to raise receives a different moral response depending on how this arrangement is described.

There are two directions in which we might explore this implication. One is to consider implications for public polling, especially where this is carried out in the service of legal reform and policy projects aiming to account for public morality. It is not a new idea that poll results will be swayed by controversial terminology or by framings utilizing jargon unlikely to be commonly used or understood. However, our results strongly suggest that the language used to characterize practically equivalent arrangements (concerning pre-conception intentions and biological parent–child relationships) influenced public moral attitudes towards those arrangements even when these terms were familiar and uncontroversial. Earlier research has indicated that prospective parents and third-party reproductive collaborators utilize language (as well as financial transactions, epistemic distance, and physical distance) to selectively emphasize certain roles and relationships to establish expectations about the moral and social parenthood of the resulting child (see e.g. Hammond 2018; Stuvøy 2018; Konrad 2005). Our study suggests that these frameworks can be extended: not only prospective parents and reproductive collaborators but the public at large view reproductive arrangements differently when these are framed in a particular way. The terminology of surrogacy and gamete donation may be understood as establishing expectations of departure from “normal” associations between biological parenthood and social parenthood—and, perhaps, legitimating this departure. This means, however, that polling projects should take into account the normative impact of such terminology, potentially by conducting parallel polls using different language and by evaluating presuppositions that any given term is genuinely applicable. As argued in (Baron 2023a) the term “double donor surrogacy” itself presupposes that “surrogacy” is an appropriate term for such arrangements; this might be a red herring.

This brings us to the second direction in which our results might be explored. From the perspective of philosophy of parenthood, we might consider whether the framing (whether terminological, clinical, or otherwise) of a reproductive/parenting agreement *does* change the moral permissibility of that agreement. The way in which many surrogacy arrangements are currently carried out means that the relevant intentions are often made manifest through negotiations, contracts, and the use of clinical intervention. On some accounts of moral parenthood, parental rights are established through the intentions of the relevant parties, reasonable expectations in light of social norms and/or the promises of others, and so on (e.g. Millum 2010; Richards 2016; Hill 1991). According to Thompson’s account of “ontological choreography,” the ways in which gametes are processed and manipulated, including the intervention of fertility clinicians, plays a role in confirming parenthood (Thompson 2005). The medical and social processes involved in surrogacy, and the expectations with which these processes are undertaken, affect which parties have a claim to the resulting child. As a result (as noted above), terms associated with assisted reproduction, such as “surrogacy” or “donor gamete,” are themselves associated with clear normative expectations regarding childrearing. Sympathy with accounts of parental rights such as those mentioned above might suggest that the terminological framing of a reproductive arrangement has an influence not only on *moral attitudes* towards it but on the *morality of the arrangement itself.* Further development of this notion is beyond the scope of this paper but would be an exciting and important avenue for future research.

### Expansion for Future Research

The hypothetical scenarios presented to participants allowed us only to gather data on attitudes to adoption/surrogacy by heterosexual couples, with names clearly associated with opposite genders. This represents a limitation to the study but one we believe is important to interpreting and communicating the results. Same-sex couples and single parents form a significant proportion of the surrogacy market. Nevertheless, they were not included in this study because we aimed to investigate differences and similarities in responses to adoption and surrogacy arrangements; there is good reason to suppose that by including homosexual and single-parent families in these randomized scenarios, the results might have been affected by biases (whether religious or political in origin) regarding “non-traditional” families. As a result, we consciously decided only to include heterosexual couples in the research to avoid any confounding influences on the data.

However, future research should assess public support for policies that allow non-traditional families to have children. Further analysis of why moral support for some policies is lower, and what factors influence this (including, as mentioned, further investigation into disparities in support for adoption/surrogacy by fertile and infertile couples), may be useful in motivating and shaping legal reform projects and policymaking. In some cases, legal changes provide the impetus for more widespread social progress. They may not have broad public support at their outset; in others, legal change may respond to what has already become a “status quo” in public morality.

As noted in section "Surrogacy and Private Adoption in U.K. Law", a further limitation of our study was in its focus on prospective parents and lack of attention to the circumstances of surrogate mothers/first families. There is a straightforward need for research on the impact of any policy reform on these groups—and indeed, the Law Commissions’ consultation report makes clear that organizations representing the interests of these groups were closely consulted in the process, which we take to be clearly positive. However, whilst public moral attitudes regarding specific practices might be relevant in informing policy reform projects, we do not believe that such research should play any significant role in shaping the legal rights of, or protections available to, surrogate mothers and first families.

## Conclusions

In light of the Law Commission’s reform project and recently published draft bill overhauling surrogacy law in the United Kingdom, there is clear and pressing need to canvass public moral support—for both new and existing policies. In this paper, we present the results of an empirical study gauging the attitudes of a representative sample of the U.K. population, with regard to adoption and surrogacy arrangements carried out by hypothetical heterosexual couples.

We found that the public expressed strong support for the current policy on surrogacy (which has been carried over into the draft bill), with 63 to 65 per cent of the public surveyed supporting the policy in cases of a fertile couple and 87 to 90 per cent supporting the policy in cases of one of the parents being infertile. We also found that the level of support for any policy (including planned private adoption, currently illegal in the United Kingdom) significantly increases if at least one of the partners experiences fertility-related issues.

That finding may suggest that people are largely sympathetic to couples experiencing trouble conceiving through unassisted procreation, though further research should be undertaken to confirm the source of this trend. Either way, future public polling projects should consider this effect, especially when aiming to gauge public morality. We argued that the same could be said for the extent to which public support can be swayed by language; such effects should likewise be considered when engaging in such research.

The findings of this study indicate some critical directions for future research, such as understanding what factors shape moral attitudes towards family-making decisions and including “non-traditional” families (such as homosexual couples and single parents) in study design to assess the effect that family type has on public support for the policy. As suggested above, however, the law should not always and only seek to reflect public morality; legal reform may sometimes play an important role in motivating public support for a policy.

## Data Availability

Data can be made available upon request.

## Footnotes

1 Whilst Krimmel expresses dismay at the idea that children might be conceived with such intentions in mind, the experiences of social workers in fostering and adoption suggest that this may not be as unimaginable as he thinks (see e.g. Moody 2018).

2 See https://www.lawcom.gov.uk/project/surrogacy/

3 Our use of a hypothetical heterosexual couple across all versions was designed to isolate—as far as possible—attitudes towards the specific adoption/surrogacy arrangements under different fertility conditions, with the aim of reducing the possibility that results would be skewed by additional biases regarding either single individuals or same-sex couples accessing adoption or surrogacy.
